# Castleman Disease in a Child: A Rare Cause of Persistent Cervical Lymphadenopathy

**DOI:** 10.7759/cureus.96352

**Published:** 2025-11-08

**Authors:** Anupam Dutta, Debajit Sarma, Pranita Medhi, Ankita Banik, Saurabh K Rabha

**Affiliations:** 1 Department of General Medicine, Assam Medical College and Hospital, Dibrugarh, IND; 2 Department of ENT, Assam Medical College and Hospital, Dibrugarh, IND; 3 Department of Pathology, Assam Medical College and Hospital, Dibrugarh, IND; 4 Department of Medicine, Assam Medical College and Hospital, Dibrugarh, IND

**Keywords:** castleman disease, cervical lymph node, histopathology, lymphadenopathy, rituximab therapy

## Abstract

Castleman disease (CD) is a rare, heterogeneous group of lymphoproliferative disorders characterized by distinctive histopathological changes. It can present as unicentric (UCD), involving a single nodal region, or multicentric (MCD), with generalized lymphadenopathy and systemic inflammation. Due to its rarity and overlapping features with infections and malignancies, diagnosis is often delayed. We report the case of a 12-year-old female who presented with progressive, multiple left-sided cervical swellings since 2023, associated with intermittent low-grade fever, headache, and fatigue. Despite empirical treatments with antibiotics and anti-tubercular therapy, her symptoms persisted. Investigations revealed anemia with hemoglobin E trait, hepatomegaly, abdominal lymphadenopathy, and minimal ascites. Cervical lymph node biopsy confirmed Castleman disease of mixed hyaline vascular and plasma cell type. Additional workup showed Epstein-Barr virus positivity, elevated IgE (>3000 IU/mL), and high IgG levels. The patient received intravenous methylprednisolone, followed by three cycles of rituximab with partial reduction of lymphadenopathy. Subsequently, a modified radical neck dissection (Type III) was performed, yielding multiple enlarged lymph nodes, the largest measuring 8 × 4 × 2 cm. Histopathology confirmed the diagnosis, showing atretic germinal centers, hyalinisation, intrafollicular vascular proliferation, and plasma cell infiltrates. This case highlights the diagnostic challenges of Castleman disease in children, its overlap with common conditions like tuberculosis, and the importance of multidisciplinary evaluation and combined medical-surgical therapy for optimal outcomes.

## Introduction

Castleman disease (CD) is a rare and heterogeneous group of lymphoproliferative disorders characterized by non-clonal proliferation of lymphoid tissue, first described by Benjamin Castleman in the 1950s [1]. CD is now recognized to encompass distinct clinical and pathological entities broadly categorized into unicentric Castleman disease (UCD) and multicentric Castleman disease (MCD) [2]. UCD typically involves a single enlarged lymph node region and is usually cured by surgical excision [2,3]. In contrast, MCD presents with generalized lymphadenopathy and systemic inflammation due to excessive cytokine production, notably interleukin-6 (IL-6) [4].

Idiopathic multicentric Castleman disease (iMCD) is a subtype of MCD in which no known viral or neoplastic trigger is identified [5]. Liu et al. conducted a systematic review of iMCD, highlighting the variable clinical presentations, ranging from constitutional symptoms and organomegaly to cytopenias and fluid accumulation [5]. Pathologically, iMCD commonly demonstrates prominent plasma cell infiltration and elevated inflammatory markers, distinguishing it from the more localized hyaline vascular variant observed in UCD [5].

Human herpesvirus-8 (HHV-8) is implicated in MCD cases associated with HIV infection, where viral IL-6 drives the cytokine storm characteristic of this disease [4]. Advances in understanding CD pathogenesis have led to the development of targeted therapies such as siltuximab, a monoclonal antibody against IL-6, which has demonstrated significant clinical benefit in iMCD [6]. International consensus guidelines now recommend anti-IL-6 therapy as first-line treatment for iMCD [7]. Despite therapeutic advances, the pathophysiology of iMCD remains incompletely understood, and ongoing research aims to elucidate its molecular underpinnings and optimize patient outcomes [3,5].

Due to its rarity and overlapping features with lymphoma and autoimmune diseases, early recognition and accurate diagnosis of Castleman disease remain essential to guide appropriate management and prevent disease-related morbidity and mortality [2,5]. 

## Case presentation

A 12-year-old female presented to the Department of Paediatrics with complaints of multiple swellings on the left side of her neck, first noted in 2023. The initial swelling was a single, mobile, non-tender lymph node approximately 1 × 1 cm in size, with a smooth surface and gradual progression in size. Over time, additional nodules developed posterior to the initial swelling, exhibiting similar characteristics. The patient also reported low-grade, intermittent fever, more pronounced in the evenings and relieved by paracetamol syrup, along with associated headache and persistent fatigue. She had no prior history of tuberculosis but gave a history of household contact with her mother, who had completed anti-tubercular therapy (ATT) for pulmonary tuberculosis in 2016.

Over the course of her illness, the patient visited multiple healthcare providers and was treated empirically with various antibiotics, including amoxicillin-clavulanic acid, and at one point received empirical anti-tubercular therapy, despite a negative Mantoux test and fine-needle aspiration cytology (FNAC) findings suggestive of reactive lymphadenitis. Ultrasonography of the neck was initially interpreted as tubercular cervical lymphadenopathy. Initial blood investigations revealed moderate anemia with a positive hemoglobin E trait, for which she received packed red blood cell transfusions. She received a total of four units of blood transfusion, one each in 2023 and 2024 during her evaluation and two units before her surgical intervention. A biopsy of the cervical lymph nodes was eventually performed, and histopathological examination was diagnostic of Castleman disease of mixed hyaline vascular and plasma cell type. Additional investigations revealed a positive IgM Epstein-Barr virus serology, markedly elevated serum IgE (>3000 IU/mL), and high IgG levels (4255 mg/dL) (Table 1). 

**Table 1:** Laboratory Blood Reports of the Patient During Hospital Stay PCV, packed cell volume; MCV, mean corpuscular volume; MCH: mean corpuscular hemoglobin; MCHC, mean corpuscular haemoglobin concentration; ANA: antinuclear antibody; SGOT, serum glutamic oxaloacetic transaminase; SGPT, serum glutamic pyruvate transaminase; AST, aspartate aminotransferase; ALT, alanine aminotransferase; Ig, immunoglobulin

Sl. No.	Parameters	Patient Values (Unit)	Reference Range (Unit)
1	HEMOGRAM		
a.	Hemoglobin	7.1 gm/dL	12–16 gm/dL
b.	Total Count	12,600 cells/µL	4,000–11,000 cells/µL
c.	Differential Count		
	• Neutrophils	72.2%	40–75%
	• Lymphocytes	19.5%	20–45%
	• Monocytes	3.3%	2–8%
	• Eosinophils	4.9%	1–6%
	• Basophils	0.1%	<1%
d.	Platelet Count	2,08,000 /µL	1,50,000–4,50,000 /µL
e.	PCV	32.8%	36–46%
f.	MCV	67.8 fL	80–96 fL
g.	MCH	19.2 pg	27–32 pg
h.	MCHC	28.4 gm/dL	32–36 gm/dL
2	HPLC	HbE trait	—
3	ANA Profile	Negative	Negative
4	KIDNEY FUNCTION TEST		
	Urea	13.20 mg/dL	10–45 mg/dL
	Creatinine	0.39 mg/dL	0.3–0.8 mg/dL
	Sodium	130.9 mmol/L	135–145 mmol/L
	Potassium	3.51 mmol/L	3.5–5.1 mmol/L
5	LIVER FUNCTION TEST		
	Total Protein	9.79 gm/dL	6.0–8.0 gm/dL
	Albumin	3.48 gm/dL	3.5–5.0 gm/dL
	Globulin	6.3 gm/dL	2.0–3.5 gm/dL
	Total Bilirubin	1.25 mg/dL	0.2–1.2 mg/dL
	SGOT (AST)	25 U/L	10–40 U/L
	SGPT (ALT)	32 U/L	7–56 U/L
	Alkaline Phosphatase	451 U/L	150–420 U/L (may be higher in children)
6	Serum Procalcitonin	0.15 ng/mL	<0.5 ng/mL
7	Serum IgG Total	4255 mg/dL	700–1600 mg/dL
8	Serum IgE	>3000 IU/L	<100 IU/L (varies with age)
9	Serum Epstein-Barr Virus IgM	Positive	Negative
10	SEROLOGY		
	• HIV	Nonreactive	Nonreactive
	• Hepatitis B	Nonreactive	Nonreactive
	• Hepatitis C	Nonreactive	Nonreactive

**Figure 1:**
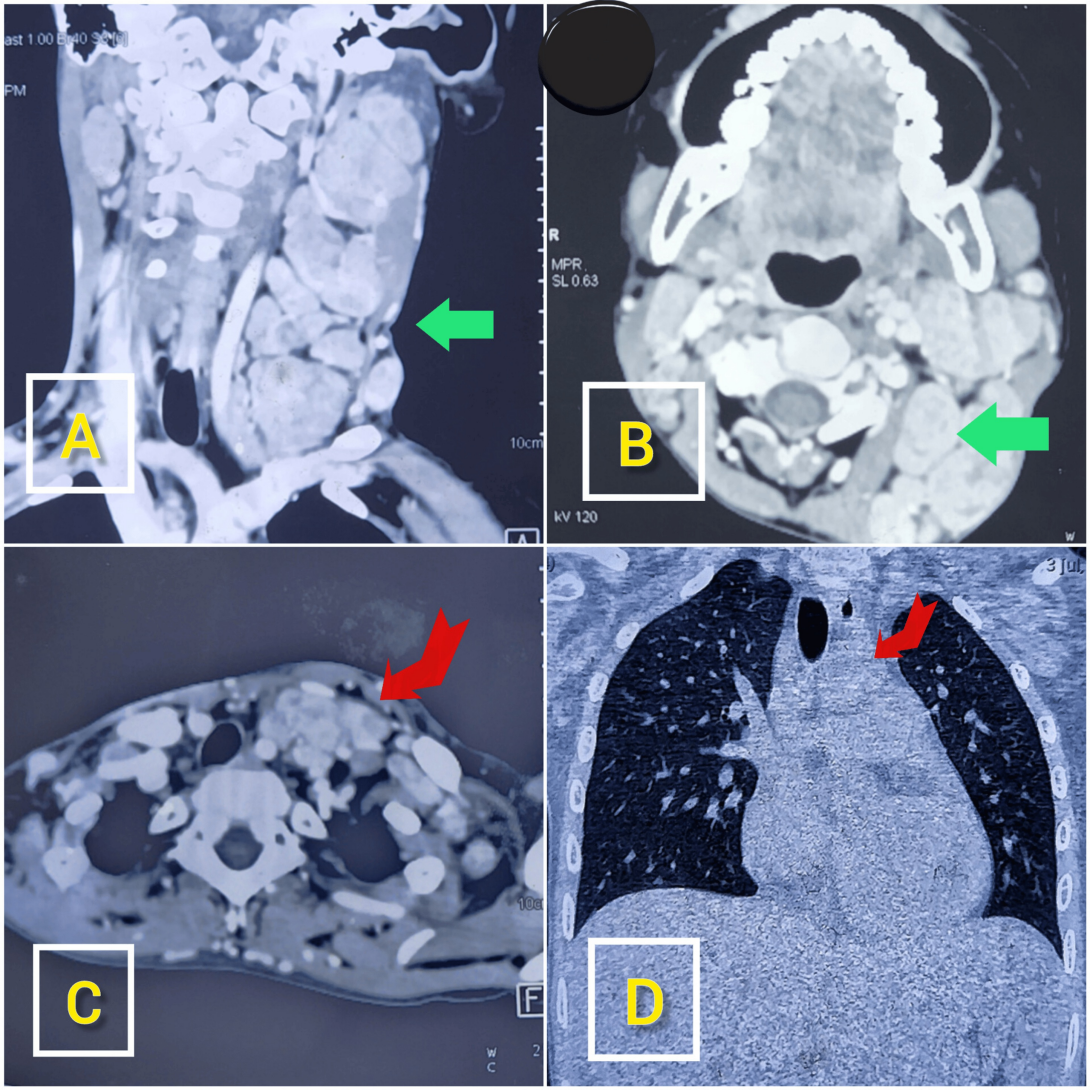
Contrast-enhanced computed tomography (CECT) Neck and Thorax showed multiple enlarged lymph nodes Multiple enlarged conglomerated lymph nodes (green arrows) in left level IB, II, III and IV in coronal section (A) and transverse section (B) of neck. Multiple enlarged lymph nodes in neck space extending up to the upper mediastinum (red angular arrows) in transverse section (C) and coronal section (D).

High-resolution CT of the neck showed multiple enlarged conglomerated lymph nodes in the left neck in various levels. High-resolution CT thorax showed multiple enlarged lymph nodes in the neck, extending into upper mediastinum (Figure 1).

During hospitalization, the patient was initially managed symptomatically with analgesics and antibiotics. After infectious causes were excluded, she was started on immunosuppressive therapy with intravenous methylprednisolone (30 mg/kg for three doses on alternate days) and was discharged on oral prednisolone. Due to persistent and progressive lymphadenopathy with systemic involvement, she was later admitted to the Department of Medicine in March 2025, where she received three monthly cycles of anti-CD20 (rituximab) infusion targeting the CD20-positive B-cell population.

**Figure 2:**
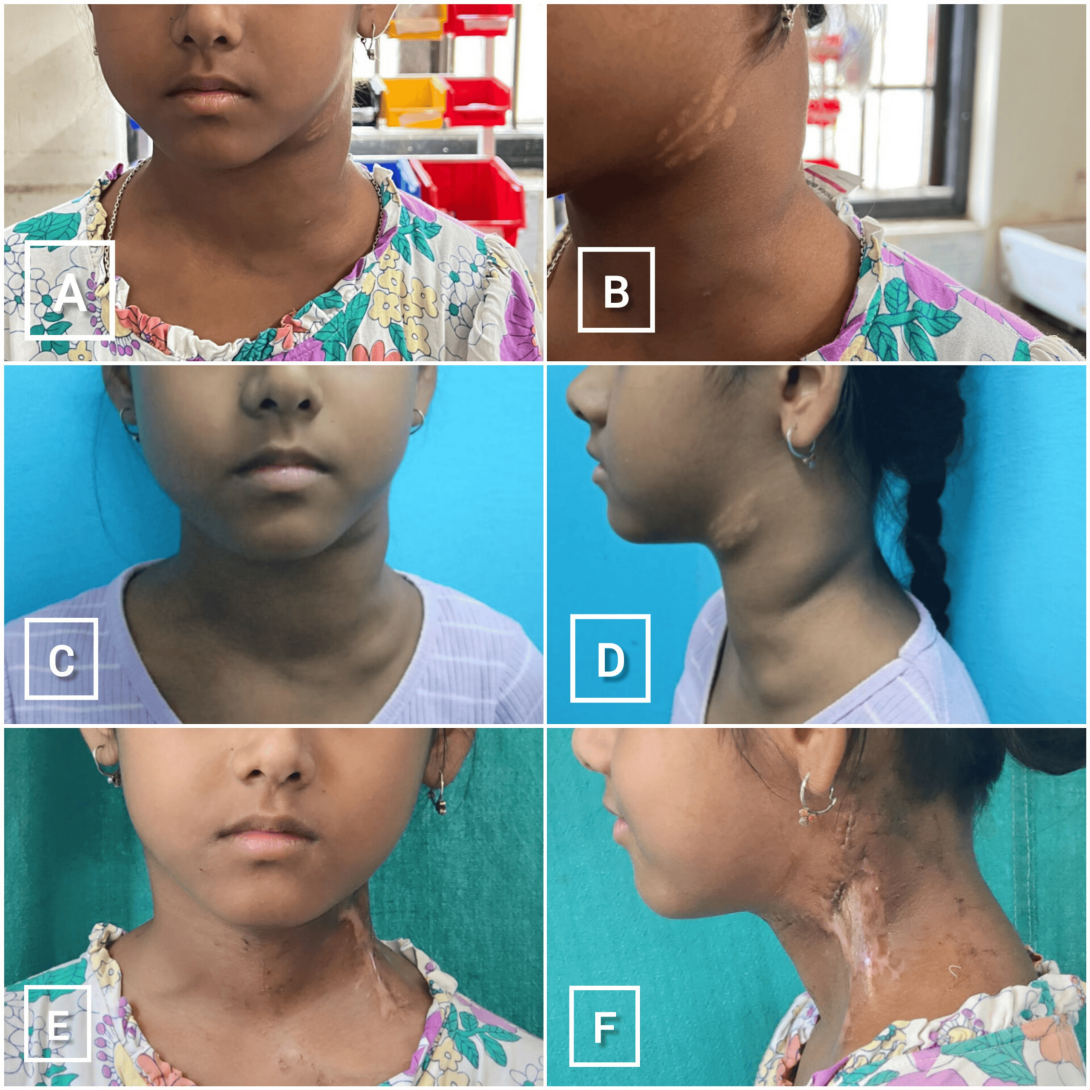
Neck swelling (cervical lymphadenopathy) due to Castelman Disease Frontal view (A) and lateral view (B) at presentation to us for evaluation, before medical therapy with steroids and rituximab. Frontal view (C) and lateral view (D) showing reduction in the size of the neck swelling after medical therapy with steroids and three monthly cycles of rituximab infusion targeting the CD20-positive B-cell population. Frontal view (E) and lateral view (F) after modified radical neck dissection (Type III).

Following medical therapy with three cycles of anti-CD20 (rituximab), the patient demonstrated a partial but significant reduction in the size of her cervical lymph nodes. However, some residual nodal masses persisted in the left cervical region. Given the incomplete resolution and associated systemic symptoms, the multidisciplinary team recommended definitive surgical intervention. Serial clinical examinations and imaging documented the gradual improvement and reduction in nodal burden, which will be illustrated with comparative images of her neck before and after therapy (Figure 2).

**Figure 3:**
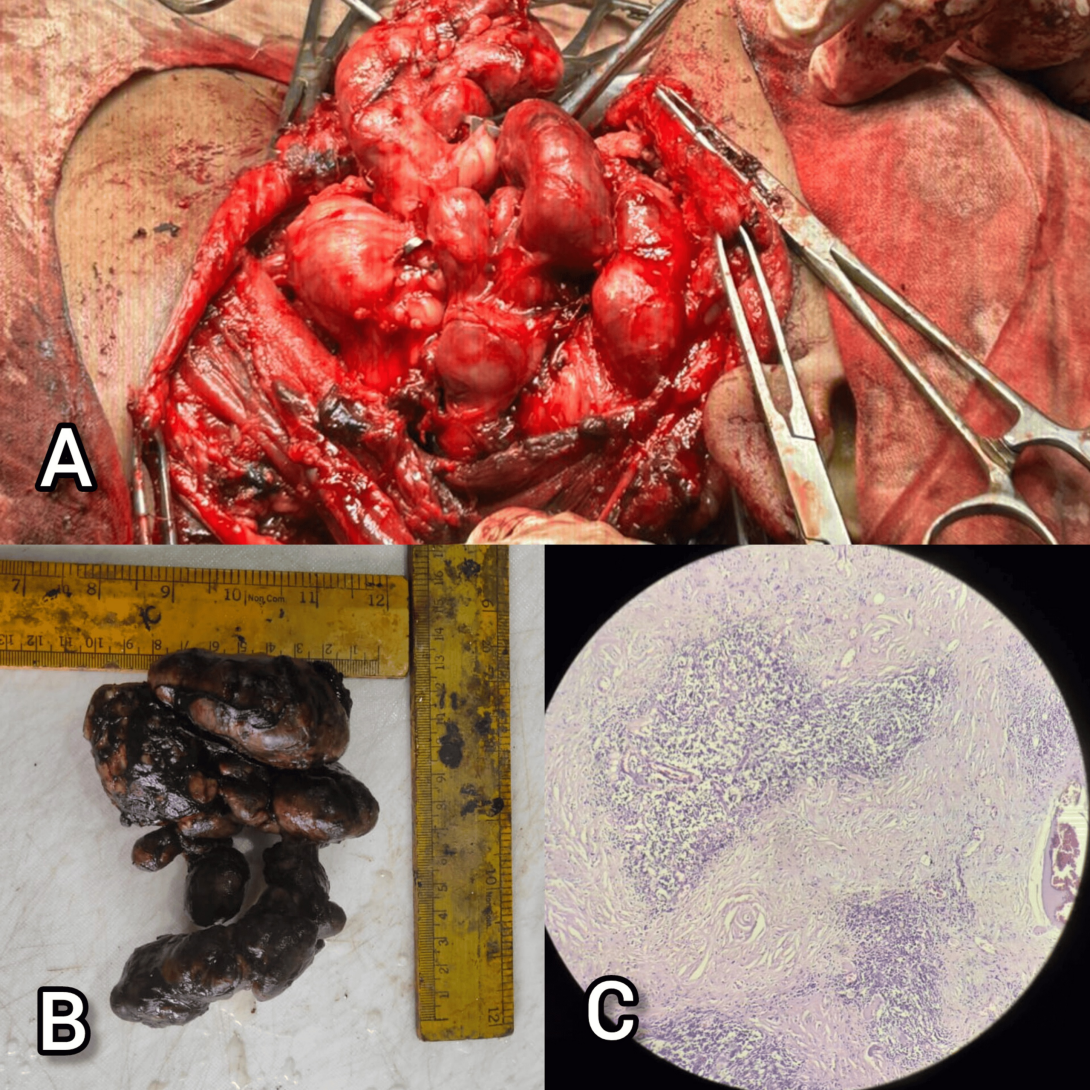
Gross and microscopic structure of the cervical lymph nodes dissected in our patient with Castleman disease A) Gross intraoperative multiple enlarged lymph nodes in the cervical region. B) Excised Multiple enlarged lymph nodes with the largest one being 8cm X 4cm X 2cm. C) Microscopic picture showing extensive areas of hyalinization with atretic follicles.(H&E, 4X)

In July 2025, the patient underwent a modified radical neck dissection (Type III) in the Department of ENT. Intraoperatively, multiple enlarged lymph nodes were noted along the left cervical chain. The largest node measured approximately 8 × 4 × 2 cm, firm in consistency, and well-encapsulated. Other smaller nodes ranging from 1 to 3 cm were also dissected out. Gross examination of the excised specimen confirmed the presence of multiple discrete lymph nodes with smooth surfaces and grey-white cut sections. The procedure was uneventful, and adequate hemostasis was achieved, with preservation of vital neurovascular structures (Figure 3).

**Figure 4:**
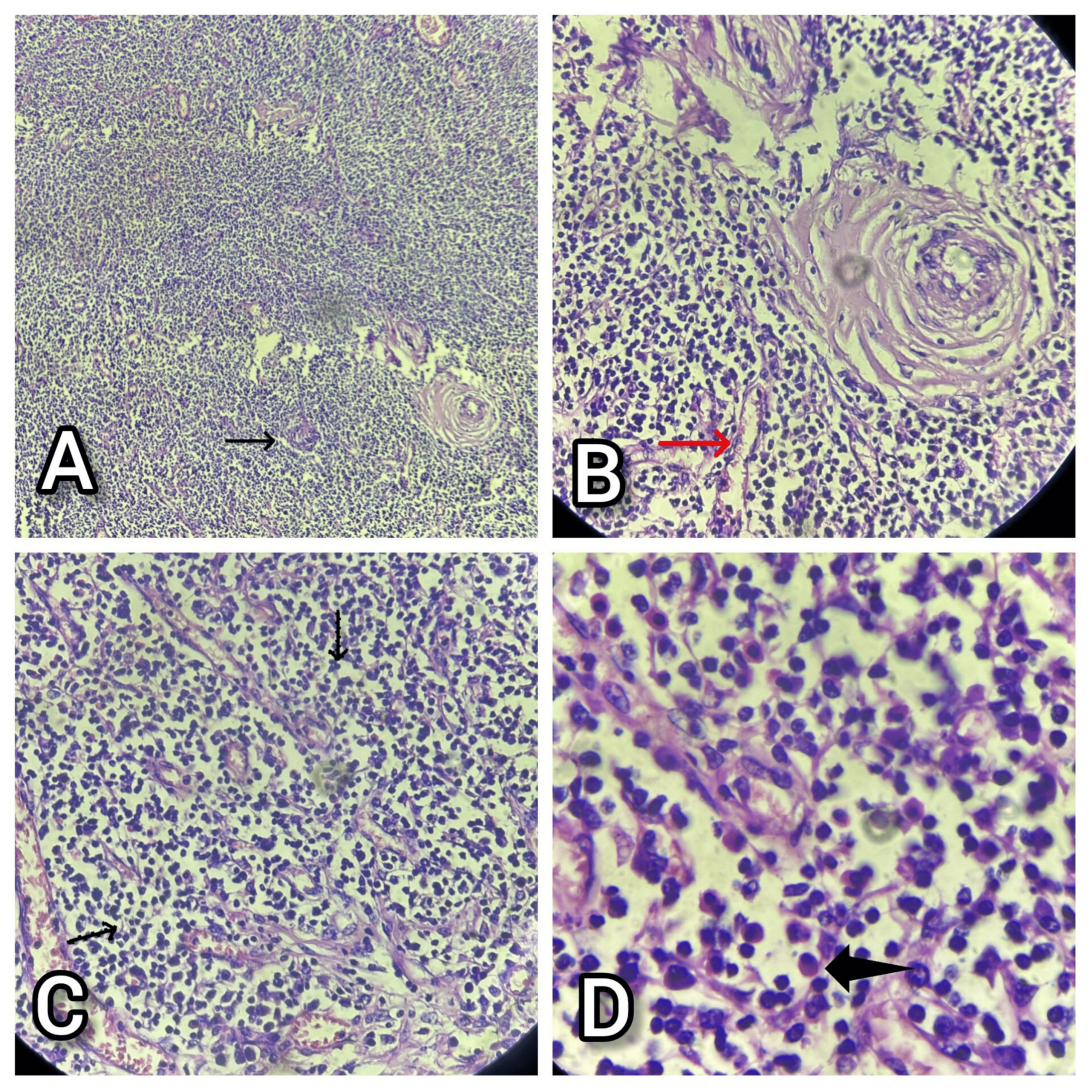
Histopathological diagnosis done to confirm the diagnosis of Castleman disease A) Atretic follicles and interfollicular vascular proliferations (black arrow) (H&E, 4X). B) Hyalinised blood vessels transgresing the follicles (red arrow) (H&E, 10X). C) Intrafollicular infiltrates (black wavy arrows) (H&E, 10X). D) Interfollicular infiltrates of plasma cells (black bold arrow) (H&E, 40X)

Histopathological examination of the resected lymph nodes confirmed the diagnosis of Castleman disease. Histologically this was compatible with hyalinized blood vessels and plasma cell subtypes. Microscopy revealed atretic germinal centers with prominent follicular dendritic cell networks and intrafollicular vascular proliferations, giving the characteristic "lollipop" appearance. Extensive hyalinisation was observed within the follicles, and there were dense interfollicular infiltrates composed predominantly of plasma cells. These features were diagnostic and corroborated the earlier biopsy findings, confirming the final diagnosis (Figure 4).

## Discussion

Castleman disease (CD), also known as angiofollicular lymph node hyperplasia, represents a rare and heterogeneous group of lymphoproliferative disorders characterized by distinctive but variable histopathological features [1]. Although originally described as a single clinicopathologic entity, it is now well established that CD comprises multiple distinct subtypes with differing etiologies, clinical presentations, and outcomes [2].

Broadly, CD is classified into UCD and MCD, based on the number and distribution of involved lymph node regions [5,2]. UCD typically presents with a localized enlargement of one or more lymph nodes in a single anatomic region, and patients are often asymptomatic or may have mild systemic symptoms due to local mass effect [8]. Surgical excision is usually curative for UCD, and prognosis is excellent, with five-year overall survival rates approaching 95% [3].

In contrast, MCD is characterized by generalized lymphadenopathy involving multiple nodal stations, accompanied by systemic inflammatory manifestations such as fever, night sweats, weight loss, hepatosplenomegaly, and laboratory features of hypercytokinemia [4]. IL-6 plays a central role in the pathogenesis of MCD, driving excessive B-cell activation, plasma cell proliferation, and systemic inflammation [7]. MCD is further subclassified based on viral association: HHV-8-associated MCD, which predominantly affects immunocompromised individuals such as people living with HIV, and iMCD, in which no underlying viral or neoplastic driver is identified [4,9]. In HHV-8-associated MCD, the virus encodes a viral IL-6 homolog that exacerbates cytokine dysregulation, explaining the striking inflammatory features in these patients [7].

Histopathologically, the hallmark of CD includes abnormal lymphoid follicles with regressed or atrophic germinal centers surrounded by expanded mantle zones arranged in concentric layers, giving an “onion-skin” appearance [8]. There is prominent proliferation of follicular dendritic cells (FDCs), increased vascularity with hyalinized vessels penetrating the follicles (“lollipop” sign), and marked interfollicular plasma cell infiltrates, especially in plasma cell and mixed variants [8,10]. Similar histologic patterns may be seen in other chronic immune activation states such as autoimmune diseases, immunodeficiencies, and certain lymphomas, which can pose diagnostic challenges [11].

Advances in understanding the pathophysiology of CD have informed new treatment strategies. For patients with iMCD, the anti-IL-6 monoclonal antibody siltuximab has shown significant efficacy and is now considered first-line therapy, as demonstrated in a pivotal phase II trial by van Rhee et al. [6]. International consensus guidelines also recommend rituximab, an anti-CD20 monoclonal antibody, for HHV-8-associated MCD, which targets the B-cell reservoir for viral replication [10]. Despite these advances, treatment remains complex in patients with refractory or severe disease, underscoring the need for continued research into the molecular mechanisms driving CD [12].

Given its rarity, varied presentations, and overlapping features with reactive lymphadenitis, tuberculosis, lymphoma, and autoimmune diseases, CD often presents a diagnostic challenge, particularly in resource-limited settings [5,10]. Timely histopathologic confirmation and subtype classification are essential to guide appropriate management and improve patient outcomes [1,9].

## Conclusions

Castleman disease, though rare and often underrecognized, presents with a wide spectrum of clinical manifestations that can mimic other lymphoproliferative or inflammatory disorders. Early recognition and accurate classification - whether unicentric or multicentric - are critical for guiding appropriate management and improving patient outcomes. This case underscores the importance of maintaining a high index of suspicion for CD in patients presenting with unexplained lymphadenopathy and systemic symptoms. Through a multidisciplinary approach involving clinical, radiological, and histopathological evaluation, timely diagnosis can be achieved. Continued awareness, research, and reporting of such cases are essential to deepen understanding and refine therapeutic strategies for this complex and heterogeneous disease.

## References

[1] Castleman B, Towne VW (1954). Case records of the Massachusetts General Hospital: case no. 40231. N Engl J Med.

[2] Fajgenbaum DC, Shilling D (2018). Castleman disease pathogenesis. Hematol Oncol Clin North Am.

[3] Talat N, Schulte KM (2011). Castleman’s disease: systematic analysis of 416 patients from the literature. Ann Surg Oncol.

[4] Oksenhendler E, Boulanger E, Galicier L (2002). High incidence of Kaposi sarcoma-associated herpesvirus-related non-Hodgkin lymphoma in patients with HIV infection and multicentric Castleman disease. Blood.

[5] Liu AY, Nabel CS, Finkelman BS (2016). Idiopathic multicentric Castleman's disease: a systematic literature review. Lancet Haematol.

[6] van Rhee F, Wong RS, Munshi N (2014). Siltuximab for multicentric Castleman's disease: a randomised, double-blind, placebo-controlled trial. Lancet Oncol.

[7] van Rhee F, Voorhees P, Dispenzieri A (2018). International, evidence-based consensus treatment guidelines for idiopathic multicentric Castleman disease. Blood.

[8] Talat N, Belgaumkar AP, Schulte KM (2012). Surgery in Castleman's disease: a systematic review of 404 published cases. Ann Surg.

[9] Fajgenbaum DC, van Rhee F, Nabel CS (2014). HHV-8-negative, idiopathic multicentric Castleman disease: novel insights into biology, pathogenesis, and therapy. Blood.

[10] Keller AR, Hochholzer L, Castleman B (1972). Hyaline-vascular and plasma-cell types of giant lymph node hyperplasia of the mediastinum and other locations. Cancer.

[11] Dispenzieri A, Fajgenbaum DC (2020). Overview of Castleman disease. Blood.

[12] Fajgenbaum DC (2018). Novel insights and therapeutic approaches in idiopathic multicentric Castleman disease. Blood.

